# Dr. Constantine Hering on Vaccination

**Published:** 1895-05

**Authors:** W. B. Clark

**Affiliations:** Indianapolis, Ind.


### DR. CONSTANTINE HERING ON VACCINATION.

W. B. Clark, M. D., Indianapolis, Ind.

Editors of the Hahnemannian Monthly:

In your February issue appears a short communication from Dr. E. M. Hale, of Chicago, headed, "A Letter from Dr. Constantine Hering," in which Dr. Hale says: "I would call the attention of Hahnemannian isopathics to his opinion of the value of varioline in small-pox." Referring to Dr. Hering's letter, we find that it was written to Dr. Hale under date of December 14th, 1871, and that his allusion to variolinum is singularly brief, he only saying: "But variolin is not sufficient in small-pox." This, after showing that sulpho-cyanates were contained both in the pus of small-pox pustules and in variolinum, thus establishing the necessary homœopathicity or isopathicity, whichever the reader may prefer.

Please permit me to call attention to another "letter from Dr. Constantine Hering" on an allied subject, written during the same decade, appearing in an English newspaper in the middle of 1878. This letter was republished by Mr. W. Young, in England, as an anti-vaccination tract, in which Dr. Hering is spoken of as the "Father of the Homœopathic School of America." In this letter Dr. Hering characterizes all vaccination as "a poisoning of the blood." Another passage reads: "In Jennerian vaccination there is the production of a real contagious disease, acting by zymosis or fermentation in the blood, thus endangering the organism." After alluding to the mix-up in the styles of vaccination, and the danger of thereby inoculating diseases, he says: "If it had been a poisoning, even with the very best real cow-pox, it now became a poisoning of nearly all children with the most horrible diseases. Many even were murdered, and an indefinite number poisoned for life." He remarked, in closing: "It is, no doubt, an intolerable tyranny to compel vaccination by law." In the language of Dr. Hale: "I would call the attention (of the whole medical profession) to his opinion of the value of vaccination, or, rather, to its dangers." As to its value, it has absolutely none, and it is a wonder that the medical profession, or any part of it, persists in hugging this superstitious delusion. It must be through ignorance. Therefore each one should stock up with the literature of the subject and peruse it—I say permit it, for it will not need study to carry conviction that vaccination does not prevent small-pox. A good example of this fact is fresh from your city in the statistical record of five thousand cases of small-pox in Philadelphia, contributed to the New York *Medical Journal*, March 17th, 1894, by Dr. Welch, which shows that at least three thousand five hundred and fifty of the cases were among vaccinated persons. As to the compulsory phase of the subject, Dr. Hering was right in stigmatizing it as "intolerable tyranny." In fact, the rock of ages on which anti-compulsory vaccination stands is that the moral sense of mankind is outraged by the official enforcement of the preposterous proposition that it is necessary to poison healthy blood to insure health; and it is this moral sense that gives each man the right to refuse to obey such criminal legislation either for himself or for his children.—*Hahnemannian Monthly*, March, 1895.

